# Serving Up FLAN. A Food Literacy and Nutrition Intervention to Fend Off Food Insecurity

**DOI:** 10.21203/rs.3.rs-4331290/v1

**Published:** 2024-05-07

**Authors:** Michael F. Royer, Michelle E. Hauser, Astrid N. Zamora, Maria Ines Campero, Dulce Garcia, Martha Gabaray, Jylana L. Sheats, Abby C. King

**Affiliations:** Stanford Prevention Research Center, Stanford University School of Medicine; Division of General Surgery, Department of Surgery, Stanford University School of Medicine; Stanford Prevention Research Center, Stanford University School of Medicine; Department of Epidemiology and Population Health, Stanford University School of Medicine; Department of Epidemiology and Population Health, Stanford University School of Medicine; Stanford Medicine Children’s Health, Lucile Packard Children’s Hospital Stanford; Tulane University School of Public Health and Tropical Medicine; Stanford Prevention Research Center, Stanford University School of Medicine

**Keywords:** food literacy, food insecurity, fruit and vegetable, nutrition, education, Latinx, Hispanic, pilot, intervention, randomized controlled trial

## Abstract

**Background:**

Food insecurity, an ongoing and accelerating problem in the U.S., is an economic and social condition involving limited or uncertain access to adequate food. Some of the highest rates of food insecurity in 2022 were found among individuals who were Hispanic-Latinx (20.8%), a population that already faces disproportionate health and socioeconomic disadvantages. There remains an urgent health-related need to identify sustainable strategies to prevent food insecurity in the Latinx population.

**Methods:**

A first-generation pilot investigation was conducted using data derived from a sub-study connected to the Computerized Physical Activity Support for Seniors (COMPASS) Trial, a 12-month cluster-randomized controlled trial among older Latinx adults. The sub-study focused on two nutrition interventions that included 1) the Food Literacy and Nutrition (FLAN) curriculum, and 2) a nutrition information-only control. Research hypotheses aimed to determine whether the FLAN intervention reduced food insecurity and increased daily fruit and vegetable servings.

**Results:**

On average, participants (n = 39) were 61.5 years of age (SD = 6.7), mostly female (69%), and reported Spanish as their primary language (69%). The FLAN intervention was associated with decreased odds of food insecurity at 12 months (AOR = 0.71, 95% CI = 0.54, 0.95; *p* = 0.03) when compared to the nutrition-information only control intervention. Although no between-group differences in daily fruit and vegetable servings were found, there was a significant correlation between changes in daily fruit and vegetable servings from baseline to six months and changes in food insecurity from baseline to 12 months (r = −0.51, *p* = 0.01).

**Conclusions:**

The FLAN intervention, a bilingual and culturally tailored educational curriculum, yielded 12-month improvements in food security among a small sample of older Latinx adults. Evidence from this investigation suggests the potential utility of implementing the FLAN curriculum among individuals who are at an increased risk of food insecurity. Further investigation in a larger sample is merited to determine whether the 12-month decreases in food insecurity that were produced by the FLAN intervention can be replicated.

**Trial Registration:**

ClinicalTrials.gov Identifier: NCT02111213

## BACKGROUND

Food insecurity, an ongoing and accelerating problem in the U.S., is an economic and social condition involving limited or uncertain access to adequate food.^1^ The prevalence of U.S. households that experienced food insecurity increased from 10.2% in 2021 to 12.8% in 2022.^2^ The highest rates of food insecurity in 2022 were found among individuals who were non-Hispanic-Black (22.4%) or Hispanic/Latinx (20.8%).^2^

With disproportionate occurrences of food insecurity comes burdensome health disparities among those who are most affected by this problem. Several unhealthy behaviors are related to food insecurity, which include physical inactivity,^3^ disordered eating,^4^ low fruit and vegetable (FV) intake,^5^ poor sleep quality,^6^ and substance misuse.^7^ Food insecurity is also associated with an increased risk of various poor health outcomes involving nutritional deficiencies,^8^ overweight and obesity,^9^ mental health problems,^10^ cardiovascular disease,^11^ and cancer.^12^ The higher-than-average food insecurity prevalence among U.S. Latinx adults presents additional burdens in the form of disease risk among a population that already faces disproportionate health and socioeconomic disadvantages.^13^

Few research interventions have been implemented to alleviate food insecurity among Latinx adults in the U.S. One quasi-experimental study examined whether a 12-week produce-prescription intervention that prescribed organic vegetables to Latinx adults with type-2 diabetes could improve food security and diabetes-related health outcomes.^14^ The produce-prescription intervention decreased the systolic and diastolic blood pressure of participants, but no significant changes were detected in their food security status. A separate quasi-experimental study tested a community garden intervention among Latinx farmworkers ages 21–78 years (n = 36) that involved gardening education, installation of a garden, and provision of gardening resources (*e.g*., seeds, tools).^15^ The gardening intervention increased both food security and vegetable intake among participants.

In another quasi-experimental study that promoted food literacy among Latinas with obesity who were either food secure or food insecure, an eight-week nutrition education program was delivered that promoted skill development in healthy food selection, healthy cooking, and financial budgeting.^16^ At the end of the program, participants who were food insecure had increased fiber-rich food intake (*i.e*., fruits, vegetables), water consumption, and physical activity levels relative to their baseline values. In addition, decreases were exhibited in sugar-sweetened beverage consumption, low-density lipoprotein cholesterol levels, body weight, waist circumference, waist-hip ratio, anxiety, and depression. However, changes in food security status were not tracked in the study. As described above, studies in this field often utilize quasi-experimental designs that have short time frames and do not include a control group. The present experimental pilot study addresses these shortcomings through random assignment of participant clusters to study treatments and a yearlong study duration.

Food literacy, which is defined by proficiency in food related skills and knowledge,^17^ is garnering increased attention as a concept with capabilities to address food insecurity. The growing consensus is that food security can be promoted by improving individuals’ food literacy through increasing their nutrition-related knowledge; developing their ability to identify, select, prepare, and cook healthy foods; and helping them navigate food-related policies and programs.^18^ The potential for food literacy to be a positive factor in sustainably improving food security deserves further systematic investigation, especially among a diverse array of disadvantaged populations given the unique social and cultural needs of people groups who are at an increased risk of food insecurity.

A small but growing number of nutrition interventions among adults have aimed to promote food security by improving specific aspects of food literacy (*e.g*., nutrition knowledge, healthy food identification, cooking skills). For example, the Nutrition Education and Skills Training (NEST) program was a six-week quasi-experimental mixed-methods study that used educational sessions with nutrition activities, goal setting, and cooking lessons to advance the food literacy of low-income Australian adults ages 18–74 years (n = 21) toward increased healthy eating and improved food security relative to their baseline values.^19^ The NEST program was successful in advancing food literacy via nutrition knowledge and cooking confidence while also achieving increases in vegetable intake and food security. A separate quasi-experimental study among college students attending a university in the southeastern U.S. involved the delivery of an 11-week educational food literacy curriculum that focused on organizing a cooking space, preparing food, reducing food waste, and cooking meals.^20^ While the intervention did not measurably improve food security, increases were detected in meal planning confidence, food preparation self-efficacy, and cooking frequency. To our knowledge, no nutrition interventions have been conducted specifically among Latinx adults in the U.S. that have promoted both food literacy and food security in this vulnerable population.

The Computerized Physical Activity Support for Seniors (COMPASS) trial was a 12-month cluster-randomized controlled trial (RCT) with a primary aim of testing the comparative effectiveness of two physical activity interventions on behavioral and health outcomes among physically inactive Latinx adults who were 50 years and older.^21^ To begin to address the current research gap concerning the relation between food literacy and food security among the Latinx adult population, COMPASS also included an experimental sub-study involving a nutrition intervention that was embedded within the larger physical activity clinical trial. The current investigation focused specifically on this nutrition intervention sub-study. COMPASS participants who engaged in the nutrition intervention sub-study received one of two programs: 1) the Food Literacy and Nutrition (FLAN) curriculum, or 2) a nutrition information-only control. The FLAN curriculum intervention group participated in a food literacy course that aimed to increase food literacy through instruction on the topics of healthy food identification, budgeting for meals, food selection, and healthful cooking. The control group, which received nutrition information only, was provided with bilingual educational pamphlets in both English and Spanish that were mailed to the participants’ homes.

For the current first-generation pilot investigation, three research hypotheses were developed to determine if either of the nutrition interventions reduced food insecurity over time. The first hypothesis estimated that the odds of food insecurity would be lower among the FLAN group than the nutrition information group at six months (the intervention adoption phase). The second hypothesis predicted that the odds of food insecurity would be lower among the FLAN group than the nutrition information group at 12 months (both the intervention adoption and maintenance phases). The third hypothesis determined whether differences in daily FV servings existed between the two study groups at the baseline, 6-month, and 12-month timepoints.

## METHODS

### Study Design

COMPASS study participants consisted of middle-age-to-older (ages 50 + years) Latinx adults who resided in the San Francisco Bay Area. Longitudinal data derived from COMPASS participants who received one of the two nutrition interventions were examined to ascertain whether the FLAN intervention produced greater improvements in food security outcomes compared to the nutrition information-only control. The Stanford University School of Medicine Institutional Review Board approved the study protocol for the COMPASS Trial. All Study materials, including informed consent and recruitment, intervention, and assessment forms, were produced in English and translated into Spanish by certified translators. Participants provided written consent to join the study upon reviewing the informed consent form with a bilingual staff member. The trial was registered at ClinicalTrials.gov (NCT02111213).

### Participant Sample

Participant eligibility criteria for the COMPASS trial required that participants be Hispanic/Latinx, 50 years of age or older, physically inactive (*e.g*., < 100 min/week moderate intensity physical activity for past six months), physically capable of engaging in moderate-intensity physical activity, and able to read and understand English or Spanish. As part of the cluster-randomized COMPASS design which involved randomization at the community center level,^21^ participants recruited from the area surrounding the Gardner Community Center in San Jose, California received the FLAN intervention, while participants recruited from the area surrounding the Dr. Martin Luther King Jr. Community Center in San Mateo, California received the nutrition information only control intervention. The participant sample for this current first-generation investigation included a total of 39 Latinx adults – 13 participants who received the FLAN intervention and 26 participants who received the nutrition information-only control intervention.

### Intervention Programs

The FLAN intervention consisted of a culturally tailored, bilingual 12-month food literacy and nutrition curriculum. Due to resource constraints, bilingual classes were delivered instead of separate English and Spanish classes. FLAN classes were held on successive Fridays at Gardner Community Center throughout the first six months of the intervention. The educational curriculum presented food literacy concepts that covered food access, local food resources, budgeting, nutritious and sustainable food choices, farm-to-table linkages, and healthy cooking. Class activities involved 1) a cooking demonstration with low-cost healthful foods by a chef who is a medical doctor and nutrition expert, 2) a potluck of meals made with traditional recipes that were modified to have healthier nutrient profiles, 3) a group visit to a nearby Grocery Outlet market to practice reading food labels and identifying and selecting healthy foods, 4) a group visit to a local farmers’ market that provided participants with $3 vouchers so they could experience how to use a Market Match^22^ program that doubled the value of their money if they spent it to purchase fruits and vegetables, and 5) the use of the Stanford Healthy Neighborhood Discovery Tool^23^ that allowed participants to collect and share information about their home and community food environments. The remaining six months of the FLAN intervention entailed a maintenance phase that included a continuation of the educational classes on a monthly basis that reinforced food literacy concepts that had been previously presented. The nutrition information-only control intervention consisted of bilingual informational pamphlets that were mailed to participants’ homes. The nutrition pamphlets consisted of basic food literacy educational information (*i.e*., healthy foods, local food resources, cooking recipes). Throughout the 12-month study period, participants in the nutrition information-only control group received the mailed nutrition pamphlets on a monthly basis.

### Outcome Measures

The primary outcome of this investigation was food security, which was measured using the U.S. Household Food Security Survey Module: Six-Item Short Form.^24^ Participants were asked to respond to six items starting with, “In the last 30 days…”, and these six items included: 1) The food that I/we bought just didn’t last, and I/we didn’t have money to get more; 2) I/we couldn’t afford to eat balanced meals; 3) Did you or other adults in your household ever cut the size of your meals or skip meals because there wasn’t enough money for food?; 4) If yes to the previous item, How often did this happen?; 5) Did you ever eat less than you felt you should because there wasn’t enough money for food?; and 6) Were you ever hungry but didn’t eat because there wasn’t enough money for food?

Food security scores were calculated using the total affirmative answers (*e.g*., Yes, Often True, Sometimes True) provided on the six items, with more affirmative answers indicating greater food insecurity. Based on the USDA’s food security scoring criteria,^24^ participants with zero affirmative answers were classified as experiencing high food security. One affirmative answer was classified as marginal food security. Two to four affirmative answers were classified as low food security. Five or six affirmative answers were classified as very low food security. The USDA’s food security scoring criteria classifies high or marginal food security as food secure and low food security or very low food security as food insecure.

Daily FV servings were measured using a self-report 60-item Nutrition Questionnaire derived from the NHANES Food Frequency Questionnaire^25^ and modified to include fruit and vegetable items that are more culturally appropriate for the Latinx population. Fourteen of the 60 items in the Nutrition Questionnaire cover the FV food group. The FV food items covered in the questionnaire include salad, tomatoes, tomato sauce, dark greens, green beans, red/orange vegetables, coconut, avocado, friend/unfried potatoes, vegetable soup, other vegetables, fresh fruit, and fruit juice. Using a Likert scale, participants indicated how frequently they had recently consumed the aforementioned FV items. Questionnaire responses were transformed into daily FV servings for each item, and a total daily FV servings variable was computed by summing the total servings across the FV items for each participant. Participant characteristics that were collected at baseline included age, sex, education, and primary language.^21^ Age was collected by asking participants how many years old they were. Sex was reported by participants indicating whether they were a biological female or male. Education was measured by participants indicating how many years of education they completed. Language was determined by participants specifying whether English or Spanish was their primary language. Intervention group was determined by the random assignment of an intervention (FLAN or nutrition information only) to one of the two community centers where participant recruitment for the two COMPASS nutrition-based interventions occurred.

### Statistical Analyses

RStudio packages including “glm” and “stats” were utilized for study data analyses.^26^ Initial analyses evaluated the relation between missing data and primary variables to determine whether the effects of the intervention and the primary outcomes of food security status at six months and 12 months were confounded by missing data. These missingness analyses assessed the relation between baseline food security status and missing data for food security status at six months and 12 months, and the relation between intervention group status and missing data for food security status at six months and 12 months.

Data for the primary study were analyzed with General Linear Models (GLM) that used bivariate regression analyses and multiple regression analyses.^27^ A an initial bivariate linear regression analysis (Model 0) first compared food security statuses between the two intervention groups. Model 0 was fitted to show whether any meaningful difference in food security status existed between the two study groups prior to the interventions. Bivariate linear regression analyses (Model 1a & Model 1b) then measured differences in food security status among the entire sample from baseline to 6 months (Model 1a) and again from baseline to 12 months (Model 1b). Model 1 analyses were fitted to show whether changes in food security status across the entire participant sample occurred throughout the study.

Unadjusted multiple linear regression analyses (Model 2a & Model 2b) assessed the effects of the interventions on food security status from baseline to 6 months (Model 2a) and from baseline to 12 months (Model 2b) with adjustment for baseline food security status but no statistical adjustment for covariates. Model 2 analyses were fitted to detect preliminary indications of the research hypotheses being supported prior to statistical adjustment for covariates as potential confounding variables. Adjusted multiple linear regression analyses (Model 3a & Model 3b) examined the effects of the interventions on food security status from baseline to 6 months (Model 3a) and from baseline to 12 months (Model 3b) while statistically adjusting for covariates. Model 3 analyses were fitted to answer the research hypotheses by determining whether the FLAN intervention outperformed the nutrition information-only intervention in improving food security over time while statistically adjusting for baseline food security status and the baseline covariates of age, sex, years of education, and primary language.

GLM’s were then used to examine between-group differences in daily FV servings among the FLAN group and the nutrition information-only control group at the baseline, 6 months, and 12 months. The first between-group comparison analyzed the relation between study intervention group (FLAN or control) and daily FV servings at baseline while statistically adjusting for the covariates of age, sex, years of education, and primary language to reduce the alternative explanations for any differences between groups at the start of the interventions. A second between-group comparison analyzed the relation between study group and daily FV servings at 6 months with similar statistical adjustment for covariates as the baseline model along with the baseline levels of daily FV servings. The final between-group comparison then analyzed the relation between intervention group status and daily FV servings at 12 months with similar statistical adjustment for covariates as the baseline and the 6-month models.

Lastly, exploratory analyses estimated the correlations between changes in food security scores (baseline to 6 months, baseline to 12 months, 6 months to 12 months) with changes in daily FV servings (baseline to 6 months, baseline to 12 months, 6 months to 12 months). These analyses explored the possibility of temporal effects of a change in one outcome (*i.e*., daily FV servings) leading to a subsequent change in another outcome (*i.e*., food insecurity). Only significant findings, if any, have been reported for these exploratory analyses.

## RESULTS

On average, participants (n = 39) were 61.5 years of age (SD = 6.7), mostly female (69%), completed 14.3 years of education (SD = 4.1), and reported Spanish as their primary language (69%) (Table 1). At six months, 31 of the original 39 participants (79.5%) were available for study assessment, including 12 of the 13 participants in the FLAN group (92.3%) and 19 of the 26 participants in the nutrition information-only control group (73.1%). By 12 months, 25 of the original 39 participants (64.1%) were available for assessment with 7 of the 13 participants in the FLAN group (53.9%) and 18 of the 26 participants in the control group (69.2%).

Across all participants, there was no association between baseline food security status and missing data at six months (B = −0.03, SE = 0.14, *p* = 0.83) or at 12 months (B = 0.13, SE = 0.16, *p* = 0.41). Baseline daily FV servings were positively associated with missing data at six months (B = 0.07, SE = 0.02, *p* = 0.01), with participants who had higher daily FV servings, regardless of group affiliation, being more likely to be lost to follow-up at six months. Across all participants, there was no association between baseline daily FV servings and missing data at 12 months (B = −0.03, SE = 0.03, *p* = 0.28). No differences were observed between the FLAN group and the control group concerning missing data for food security status at six months (B = −0.19, SE = 0.14, *p* = 0.17) or at 12 months (B = 0.15, SE = 0.17, *p* = 0.36).

### Changes in Food Insecurity

No between-group differences in food security status (Model 0) were present among the FLAN group and the control group at baseline (B = −0.15, SE = 0.17, *p* = 0.37) (Table 2). Across all participants, the odds of experiencing food insecurity increased from baseline to six months (Model 1a) (OR = 1.45, 95% CI = 1.06, 1.99, *p* = 0.03), but this increase was attenuated by the 12-month timepoint (Model 1b) (OR = 1.38, 95% CI = 0.98, 1.93; *p* = 0.08). Unadjusted between-group analyses revealed no differences in the odds of experiencing food insecurity from baseline to six months (Model 2a) (AOR = 1.07, 95% CI = 0.77, 1.49, *p* = 0.68) nor from baseline to 12 months (Model 2b) (AOR = 0.74, 95% CI = 0.52, 1.05, *p* = 0.10).

**Figure 1:**
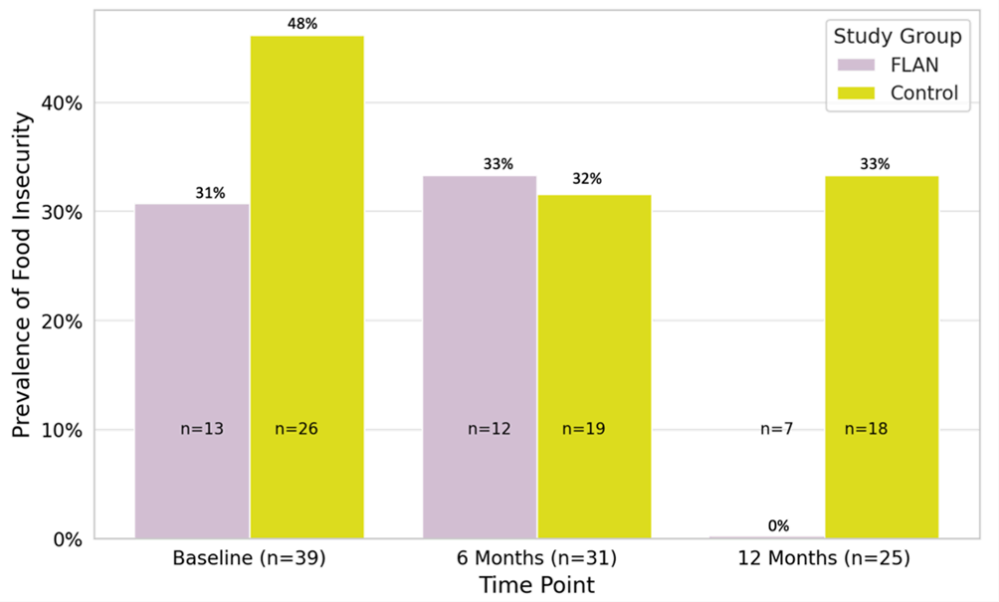
Prevalence of Food Insecurity Over Time by Study Group

Adjusted between-group analyses produced findings that supported those from the previous model that showed no differences in the odds of experiencing food insecurity from baseline to six months (Model 3a) (AOR = 1.08, 95% CI = 0.78, 1.49, *p* = 0.64). In contrast, the final model that adjusted for covariates indicated that the FLAN intervention reduced the odds of food insecurity from baseline to 12 months (Model 3b) (AOR = 0.71, 95% CI = 0.54, 0.95; *p* = 0.03) in comparison to the control group. The FLAN intervention effects on food insecurity are depicted in Fig. 1.

The between-group comparisons that assessed differences in daily FV servings by study group showed no differences throughout the study (Table 3). At baseline, the mean daily FV servings was 2.8 (SD = 2.0) for the FLAN group and 4.1 (SD = 2.8) for the nutrition information-only control group (*p* = 0.84). After six months, the mean daily FV servings was 4.3 (SD = 2.3) for the FLAN group and 3.5 (SD = 2.2) for the control group (*p* = 0.41). At 12 months, the mean daily FV servings was 4.8 (SD = 2.2) for the FLAN group and 4.7 (SD = 2.7) for the control group.

Lastly, exploratory correlation analyses among the entire sample evaluated the extent to which changes in daily FV servings were associated with changes in food insecurity by 12 months. One exploratory analysis detected a significant negative correlation between changes in daily FV servings from baseline to six months and changes in food insecurity from baseline to 12 months (r = −0.51, *p* = 0.01).

## DISCUSSION

This first-generation pilot study evaluated the initial efficacy of a new intervention, called FLAN, in promoting food literacy in older Latinx adults from low-income neighborhoods. Study outcomes suggest that the FLAN intervention reduced the odds of food insecurity at 12 months in comparison to the nutrition information-only control group. The significant effects observed for the FLAN intervention on food insecurity at 12 months, but not at 6 months, suggest that changes in food literacy toward improved food security in this older Latinx population may take time to occur. Despite FLAN not having a significant effect on daily FV servings at 6 months or 12 months, outcomes from follow-up exploratory analyses indicated that increases in daily FV servings from baseline to 6 months were correlated with decreases in food insecurity from baseline to 12 months.

The FLAN intervention delivered a variety of educational classes combined with hands-on activities to promote food literacy by helping participants identify, prepare, and cook healthy foods that were culturally tailored for this sample. Hands-on and culturally relevant activities were integrated into the curriculum, which included healthy cooking practices (*e.g.,* adding vegetables, minimizing sodium, etc.) and navigating the local food landscape through trips to a local farmers’ market, food bank, and grocery store. Prior to the start of the study, investigators and staff trained in nutrition, including instructors from the Latinx community, participated in developing and pre-testing portions of the FLAN intervention to ensure its relevance and acceptability for this population. Based on observations reported by study staff throughout the intervention, the FLAN curriculum content was found to be well accepted by participants, as indicated by participants’ self-reported uptake in using ingredient lists on packages to make healthy choices when shopping and their reports during the class sessions that detailing their agreement of it being easy to make healthy dietary changes while maintaining cultural food traditions.

Previously developed community partnerships with local organizations, including a farmers’ market, a food bank, and a grocery store, fostered collaborative learning experiences that allowed participants to directly apply what they had learned during their FLAN classes in their own community. As a result of what they learned from the FLAN curriculum, participants started bringing food items to classes to share with others and communicating the healthy changes that they made to various traditional recipes to increase their healthfulness. These health-enhancing recipe modifications were put on full display at group potlucks that occurred during class time.

At six months into the FLAN curriculum, the lack of discernable effects of the intervention on food security may have been due to the observation that participants were still in the process of learning and applying their newly developed skills. This could explain why food security was shown to have improved at 12 months, but not at six months, as the benefits of the FLAN intervention (*e.g*., knowledge, skills, resources) needed sufficient time to make meaningful improvements to food security. A prominent highlight from the study is how no remaining FLAN intervention group participants reported being food insecure at 12 months. Separately, it appeared that the FLAN intervention did not impact daily FV servings, as there were no between-group differences detected for daily FV servings at the 6-month or 12-month timepoints. The mean average daily FV servings for this study sample seemed to cumulatively meet the 2020–2025 Dietary Guidelines for Americans that provides recommendations for adults to consume one-and-a-half to two cups of fruits and two to three cups of vegetables daily.^28^ Furthermore, the significant negative correlation between changes in daily FV servings from baseline to six months and changes in food insecurity from baseline to 12 months hints at the possibility of non-significant changes in daily FV servings still having a meaningful effect on successive changes in food security status from baseline to 12 months. Such reflections await further evaluation in larger samples of Latinx adults.

Outcomes from the FLAN intervention shared some similarities with other research interventions in improving food security among Latinx adults. A two-year community gardening project that was part of a quasi-experimental study among Latinx adults ages 21–78 years (n = 36) demonstrated improved food security by reducing the monthly frequency of participating families’ concerns about running out of food before having enough money to buy more.^15^ The gardening intervention outcome, like those from FLAN, included significant pre-post improvements in food security from baseline to ≥ 12 months. A major contrast between the FLAN and gardening intervention was how the gardening project increased FV intake over time while FLAN did not achieve that outcome. The NEST program, which was a quasi-experimental study among Australian adults ages 18–74 years (n = 21), shared several similarities with the FLAN intervention. Like FLAN, the NEST study involved a small participant sample, promoted food literacy with its intervention, had a pre-post design, and yielded improvements in food security.^19^ Moreover, a systematic review of research articles detailing the relation between food insecurity and dietary quality among U.S. adults and/or children (n = 26) determined that U.S. adults who were food insecure consumed significantly less FVs than their food secure counterparts.^29^ These findings align with results from the present study that highlight the significant negative correlation between changes in daily FV servings and ensuing changes in food security status.

### Strengths & Limitations

This study contained several strengths. During the development of the FLAN intervention, a preliminary focus group and related community exploratory activities were conducted among older Latinx adults in the surrounding area to determine what types of healthy food items are most appealing and available to them. These preliminary activities enhanced the tailoring process of the intervention curriculum by ensuring the applicability and accessibility of the informational content. The bilingual curriculum offering was another strength of FLAN, as a majority of participants reported Spanish as their primary language. Producing and delivering the curriculum in both English and Spanish increased the accessibility of FLAN by allowing individuals to receive the benefits of the program who might have otherwise been unable to participate due to a language barrier. The cluster-RCT study design strengthened the validity of the research findings by randomly allocating the two interventions to two local community center sites, which allowed for between-group comparisons of food security status to be made throughout the study. Statistical tests for missingness indicated that there were no significant differences in attrition between the intervention and control groups. It also appeared that the FLAN intervention’s effect on food security status was not unduly impacted by participant attrition. Although, significant differences in data missingness were detected at 6 months among participants with higher baseline daily FV servings, but these differences became non-significant at 12 months.

Limitations were also present in this study. This first-generation research included a small participant sample, which resulted in reduced statistical power to detect intervention effects. The increases in food security that were detected provide an encouraging first step in this line of research by indicating that a larger-scale study would be worthwhile to pursue. The small sample size and subsequent participant attrition throughout the study, due primarily to resource constraints for this sub-study, underscore the need to interpret both significant and non-significant study findings with caution. Analyses that evaluated the impacts of missing data on the primary study outcomes suggested that no differences in data missingness existed by baseline food security status nor by study group. However, participants with missing data for daily FV servings at 6 months significantly differed from those with complete data for baseline daily FV servings, which suggests that the findings for the effect of the intervention on daily FV servings at 6 months were potentially confounded by the absence of the data from the participants who were lost to follow up. The inclusion of older Latinx adults living in the San Francisco Bay Area in this pilot research reduced the external validity of study findings by limiting their generalizability to adults of different age groups, races and ethnicities, and from other regions of the country. This limitation could be attenuated by replicating the present study and establishing culturally tailored food literacy curricula for other U.S. Latinx/Hispanic groups as well as additional racial and ethnic groups. Nevertheless, it is important to recognize the large amount of evidence supporting health promotion interventions that are customized for specific demographic groups, as opposed to attempts to utilize “one size fits all” interventions which often do not meet the needs and references of different population segments.^30^

## CONCLUSIONS

The FLAN intervention, a bilingual and culturally tailored educational curriculum, aimed to increase the food literacy of Latinx adults by teaching concepts involving budgeting, food choices, and healthful cooking. Evidence from this initial research investigation emphasizes the promise of the FLAN curriculum in promoting food security among the Latinx population, which faces an increased risk of food insecurity and chronic diseases related to insufficient access to healthy foods. The results from this pilot study support further investigations to more comprehensively determine the effectiveness of applying this type of interactive food literacy intervention to alleviate and prevent food insecurity.

## Figures and Tables

**Table 1:** Baseline Characteristics of Latinx Adult Participants who received a Nutrition-basedCOMPASS Intervention (n = 39)

Characteristics	Full Sample (%)	FLAN (%)	Control (%)
*Age (Years)*	M = 61.5, SD = 6.7^a^	M = 61.8, SD = 5.1	M = 60.7, SD = 7.4
*Sex*			
Female	27 (69)	8(62)	19(73)
Male	12 (31)	5(38)	7(27)
*Education (Years)*	M = 14.3, SD = 4.1	M = 14.0, SD = 3.6	M = 14.2, SD = 4.5
*Primary Language*			
English	12 (31)	3(23)	9(35)
Spanish	27 (69)	10 (77)	17(65)
*Food Security Status*			
Food Secure	23 (59)	9(69)	14(54)
Food Insecure	16 (41)	4(31)	12(46)
*Daily FV*^a^ *Servings*	M = 3.9, SD = 2.5	M = 3.7, SD = 2.0	M = 4.1, SD = 2.8
*Intervention*			
FLAN	13 (33)		
Nutrition Information	26 (67)		

a FV = Fruit & Vegetable; M = Mean; SD = Standard Deviation

**Table 2:** General Linear Models Analyzing the Effect of the FLAN Intervention on Food Insecurity among LatinxAdults (n = 39)

GLM Model	Coefficient	Odds Ratio	95% CI	*p*-Value
*Model 0 (BL)*	B = −0.15, SE = 0.17			0.37
*Model 1a (BL◊6M)*	**B = 0.37, SE = 0.16**	**OR = 1.45**	**1.06,1.99**	**0.03**
*Model 1b (BL◊12M)*	B = 0.32, SE = 0.17	OR = 1.38	0.98,1.93	0.08
*Model2a (BL◊6M)*	B = 0.07, SE = 0.17	AOR = 1.07	0.77,1.49	0.68
*Model2b (BL◊12M)*	B = −0.30, SE = 0.18	AOR = 0.74	0.52,1.05	0.10
*Model3a (BL◊6m)*	B = 0.08, SE = 0.16	AOR = 1.08	0.78,1.49	0.64
*Model3b (BL◊12m)*	**B = −0.34, SE = 0.15**	**AOR = 0.71**	**0.54,0.95**	**0.03**

a Model 0 = between-groups comparisons of food security status at baseline

b Models 1a & 1b = pre-post odds of food insecurity among entire sample from baseline to six-monthsand baseline to 12-months

c Models 2a & 2b = between-groups comparisons for odds of food insecurity from baseline to six-months and baseline to 12-months with adjustment for baseline food security status but not forcovariates

d Models 3a & 3b = between-groups comparisons for odds of food insecurity from baseline to six-months and baseline to 12-months with adjustment for baseline food security status and covariates

e 6M = 6 months; 12M = 12 months; AOR = adjusted odds ratio; B = unstandardized beta coefficient; BL = baseline; CI = confidence interval; GLM = general linear model; OR = odds ratio; SE = standarderror

**Table 3:** Between-Group Comparisons of Daily Fruit & Vegetable Servings and Food InsecurityPrevalence Over Time among Latinx Adults (n = 39)

Outcome	Time Point	FLAN	Control	*p*-Value
*Daily FV*^a^ *Servings*				
	Baseline	M = 2.8, SD = 2.0	M = 4.1, SD = 2.8	*p* = 0.84
	6 Months	M = 4.3, SD = 2.3	M = 3.5, SD = 2.2	*p* = 0.41
	12 Months	M = 4.8, SD = 2.2	M = 4.7, SD = 2.7	*p* = 0.52
*FI*^a^ *Prevalence*				
	Baseline	31%	48%	*p* = 0.28
	6 Months	33%	32%	*p* = 0.89
	12 Months	**0%**	**33%**	**p = 0.03**

a FI = Food Insecurity; FV = Fruit & Vegetable; M = Mean; SD = Standard Deviation

b All between-group comparison analyses used General Linear Models to regress the primary outcomevariable at each timepoint on the intervention group variable with statistical adjustment for thecovariates of age, sex, years of education, and primary language.

## Abbreviations

FV: Fruit and Vegetable
NEST: Nutrition Education and Skills Training
COMPASS: Computerized Physical Activity Support for Seniors
RCT: Randomized Controlled Trial
FLAN: Food Literacy and Nutrition
NHANES: National Health and Nutrition Examination Survey
GLM: General Linear Model
B: Beta coefficient
SE: Standard Error
M: Mean
SD: Standard Deviation
CI: Confidence Interval
FI: Food Insecurity
